# Tubulointerstitial nephritis with monotypic lympho-plasmacytic infiltrates in a patient with primary Sjögren’s syndrome accompanied by IgA-type monoclonal gammopathy

**DOI:** 10.1186/s12882-019-1646-x

**Published:** 2019-12-12

**Authors:** Takako Saeki, Takashi Kuroha, Yuya Sato, Maasa Tamura, Akira Iguchi, Tomoyuki Ito, Hajime Yamazaki, Yumi Ito, Kazuhiro Yoshita, Naofumi Imai, Ichiei Narita, Hiroyuki Usuda

**Affiliations:** 1 Department of Internal Medicine, Nagaoka Red Cross Hospital, 2-297-1 Senshu, Nagaoka, Niigata, 940-2085 Japan; 2 Division of Clinical Nephrology and Rheumatology, Niigata University Graduate School of Medical and Dental Sciences, 1-757 Asahimachi-dori, Niigata, 951-8510 Japan; 3 Department of Pathology, Nagaoka Red Cross Hospital, 2-297-1 Senshu, Nagaoka, Niigata, 940-2085 Japan

**Keywords:** Tubulointerstitial nephritis, Monotypic lympho-plasmacytic infiltrates, Monoclonal gammopathy of undetermined significance, IgA paraproteinemia, Primary Sjögren’s syndrome

## Abstract

**Background:**

Although most cases of tubulointerstitial nephritis in paraproteinemia are monoclonal light chain deposition-mediated, interstitial nephritis as neoplastic interstitial cell infiltration has rarely been described. On the other hand, lympho-plasma-cell-rich tubulointerstitial nephritis, in which the infiltrative cells are usually polytypic, is often evident in primary Sjögren’s syndrome (pSS). Herein we present a rare case of pSS in a patient who had been diagnosed as having IgA kappa-type monoclonal gammopathy of undetermined significance (MGUS) and developed tubulointerstitial nephritis with monotypic (IgA kappa) lympho-plasmacytic infiltrates.

**Case presentation:**

A 74-year-old Japanese woman with pSS who had been diagnosed as having IgA kappa-type MGUS developed progressive renal dysfunction. Renal biopsy revealed tubulointerstitial nephritis with abundant plasma cell-rich mononuclear cell infiltrates without atypia. Immunohistochemical staining for immunoglobulins and light chains showed that most infiltrates were positive for IgA and kappa. Most of the infiltrative cells were positive for CD38 and CD138, and cells positive for CD 19 and CD 45 were also widely evident. Electron microscopy and immunofluorescence studies revealed no apparent immunological deposits in the glomeruli and tubules. Bone marrow and whole-body radiological examinations revealed no findings suggestive of multiple myeloma or lymphoma. Renal function improved rapidly with prednisolone 40 mg daily and has been maintained at the same level on low-dose prednisolone and azathioprine for 18 months.

**Conclusion:**

Tubulointerstitial nephritis with monotypic cell infiltrates, without immunological deposits, is a quite rare histological picture in MGUS, and might be a unique renal manifestation in patients with pSS.

## Background

Kidney disease is a frequent complication of paraproteinemia, including multiple myeloma or B-cell lymphoma [1, 2]. Although light chain cast nephropathy is the most frequent form of renal injury in multiple myeloma, deposition of secreted monoclonal immunoglobulin or a fragment thereof has been shown to occur even in patients with B-cell clones that do not meet the definition of multiple myeloma or lymphoma, i.e. monoclonal gammopathy of undetermined significance (MGUS) [1, 3]. Traditionally, MGUS has been considered a relatively benign entity with a low likelihood of progression to myeloma, and chemotherapy is not usually indicated. However, it has been recognized that the renal disorders caused by monoclonal immunoglobulin can sometimes be associated with severe and irreversible renal injury, and the term “monoclonal gammopathy of renal significance” (MGRS) has recently been proposed [1, 3]. Recently, the term MGRS has been applied specifically to any B cell or plasma cell clonal lymphoproliferation with both of the following characteristics: 1) One or more kidney lesions that are related to the produced monoclonal immunoglobulin. 2) The underlying B cell or plasma cell clone does not meet any current hematological criteria for specific therapy [4]. Here we present a rare case of pSS in a patient who had been diagnosed as having IgA-type MGUS and developed tubulointerstitial nephritis with monotypic (IgA-kappa) lympho-plasmacytic infiltrates.

## Case presentation

A 74-year-old Japanese woman with pSS accompanied by MGUS was admitted to our hospital due to progressive renal dysfunction and interstitial lung disease. The patient had no family history of renal diseases. She had been diagnosed as having pSS 16 years previously, based on the objectively confirmed presence of dry eyes, dry mouth and positivity for anti-Ro/SSA and anti-La/SSB antibodies. Because she had no extraglandular organ involvement at that time, she had received local treatment for the ocular and oral symptoms. Four years after the diagnosis of pSS, bilateral lung reticular shadows were noticed in a radiological examination, along with elevation of the KL-6 titer. Although she was diagnosed as having interstitial lung disease-associated pSS based on the results of high-resolution computed tomography and bronchoalveolar lavage studies (increased lymphocytes without any findings suggestive of infection or malignancy), the reticular shadows and elevated serum KL-6 level improved spontaneously and no respiratory symptoms became evident, and therefore she had been followed up without steroid therapy. Six years before presentation, she had developed salivary gland swelling and was treated with prednisolone 10 mg daily in the short term, being maintained thereafter with low-dose prednisolone (2 mg daily). From 3 years before presentation, the serum level of IgA had gradually increased, along with a converse decrease of the serum IgG level. Serum protein electrophoresis demonstrated M-protein, and immunofixation revealed that the M-protein was the IgA-kappa type. The serum free light chain kappa/lambda ratio was also elevated. Plasma cells in the bone marrow accounted for 5% of the total, and no bone lesions or hypercalcemia were evident. The patient was therefore diagnosed as having pSS with MGUS (IgA-kappa) and was maintained on low-dose prednisolone therapy (2 mg daily). By 8 months before admission, the serum level of creatinine had been almost stable at 0.7–0.75 mg/dl [estimated glomerular filtration rate (eGFR) 61.6–57.0 ml/min/1.73^2^]. However, the level gradually increased thereafter, and was 1.2 mg/dl (eGFR 34.0 ml/min/1.73^2^) at the time of admission. In addition, the bilateral lung reticular shadows worsened during the few months before admission, and a dry cough had developed.

On admission, the patient was 142 cm tall and weighed 47 kg, with a blood pressure of 120/80 mmHg. She was afebrile and showed no abnormal physical findings except for severe xerostomia. Mild bibasilar crackles in the lung were noted. Urinalysis showed pH 6.5, no hematuria, a scanty urinary sediment, and mild proteinuria (0.2 g/day). Glucosuria was not evident. The hemoglobin level was 9.6 g/dl, white blood cell count 4670/μl and platelet count 271,000/μl. The serum creatinine level was 1.20 mg/dl (normal range; 0.40–0.80), eGFR 34.0 ml/min/1.73^2^, and blood urea nitrogen 31.6 mg/dl (8.0–22.0). Serum electrolytes, liver function data and HbA1c were within the normal ranges. The serum KL-6 level was 1171 U/ml (normal < 500). The serum C-reactive protein level was 0.05 mg/dl, the serum total protein level 8.7 g/dl, albumin 3.9 g/dl, IgG 1092 mg/dl (normal range; 870–1700), IgA 3144 mg/dl (110–410) and IgM 82 mg/dl (57–288). The serum CH50 level was 68 U/ml (normal range; 32–58). The serum concentration of free kappa light chain was 89.3 mg/dl (normal range; 2.42–18.92), lambda 27.60 mg/dl (4.44–26.18) and the kappa/lambda ratio 3.236 (0.244–1.804). Urinary excretion of N-acetyl-β-glucosaminidase and urinary β2-microglobulin was markedly elevated to 42.9 U/day (normal < 10 U/day) and 10.362 mg/day (normal < 0.4 mg/day), respectively. Blood gas analysis demonstrated no metabolic acidosis (pH 7.377, HCO3 23.4 mmol/l). A 24-h urine collection for electrophoresis and immunofixation showed no paraprotein. Plasma cells in the bone marrow accounted for 3.2% of the total. Whole-body CT examination revealed basilar reticular opacities in both lungs, but no lytic bone lesion or tumorous lesion. ^18^FDG-PET/CT scanning showed FDG uptake only in the bilateral lungs.

**Fig. 1:**
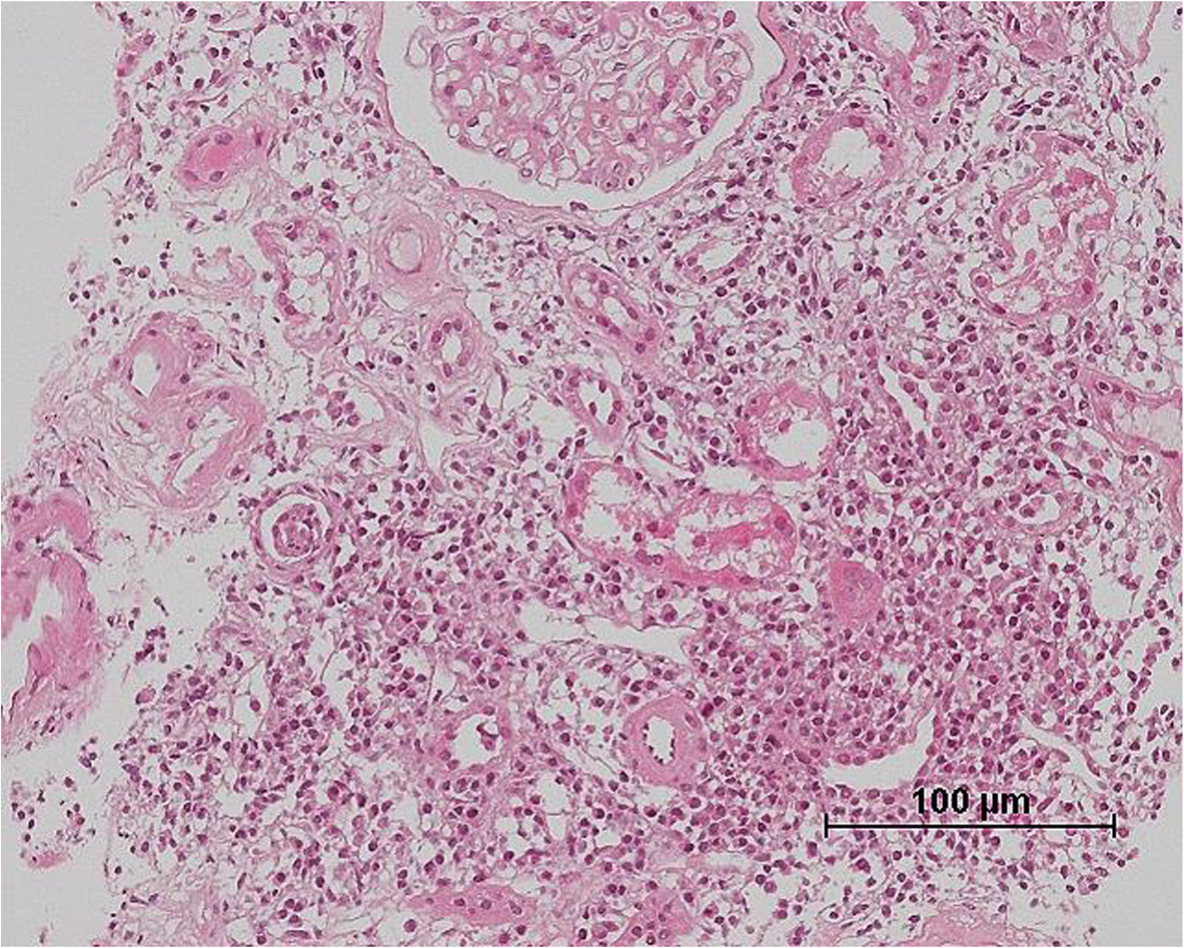
Light microscopy findings in the renal tissues. Diffuse marked renal interstitial inflammation consisting of plasma cell-rich mononuclear cells is demonstrated. (Hematoxylin and eosin stain, × 200)

**Fig. 2:**
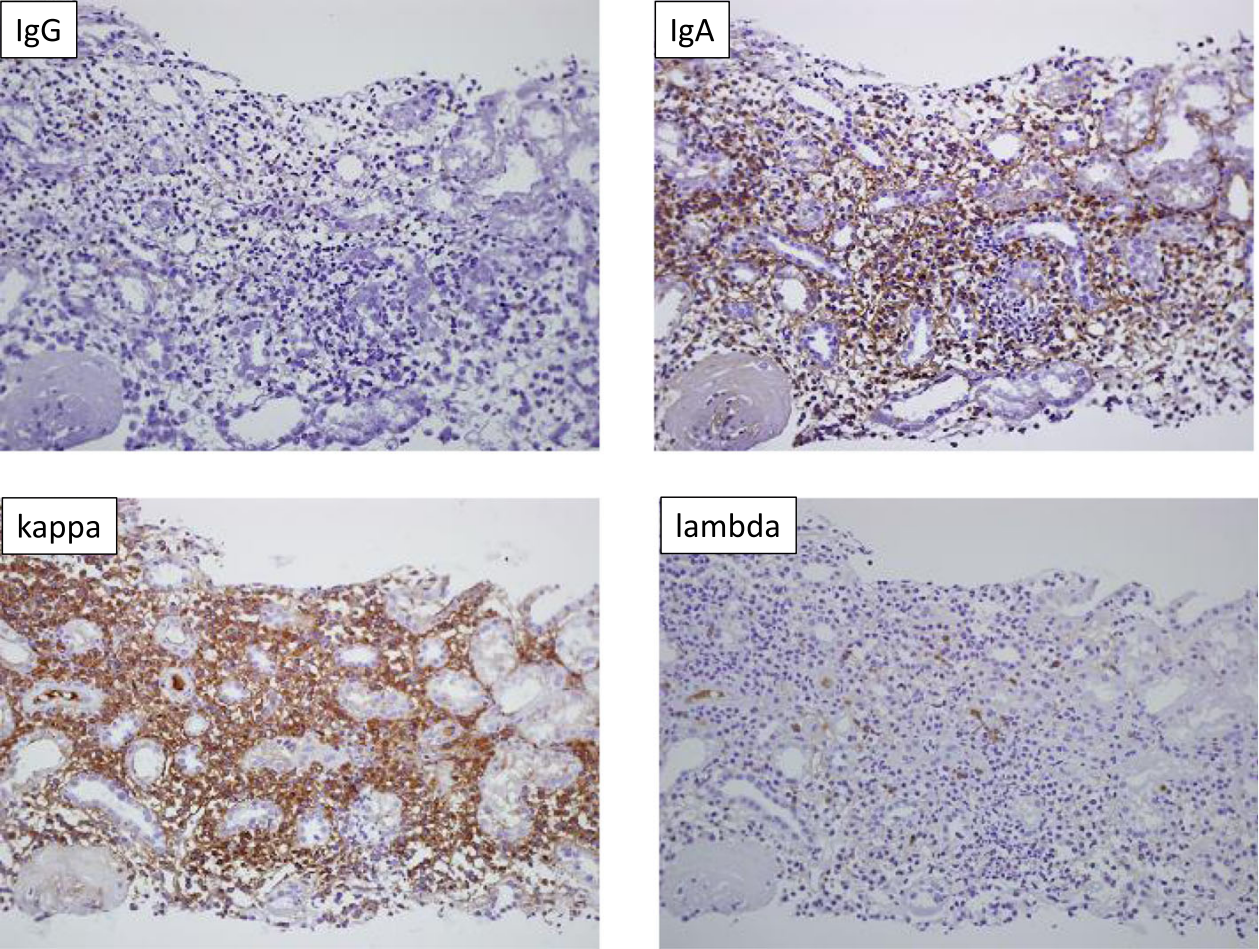
Immunohistochemical staining for IgG, IgA, kappa and lambda of renal biopsies (× 200). Most of the infiltrative cells were positive for IgA and kappa

**Fig. 3:**
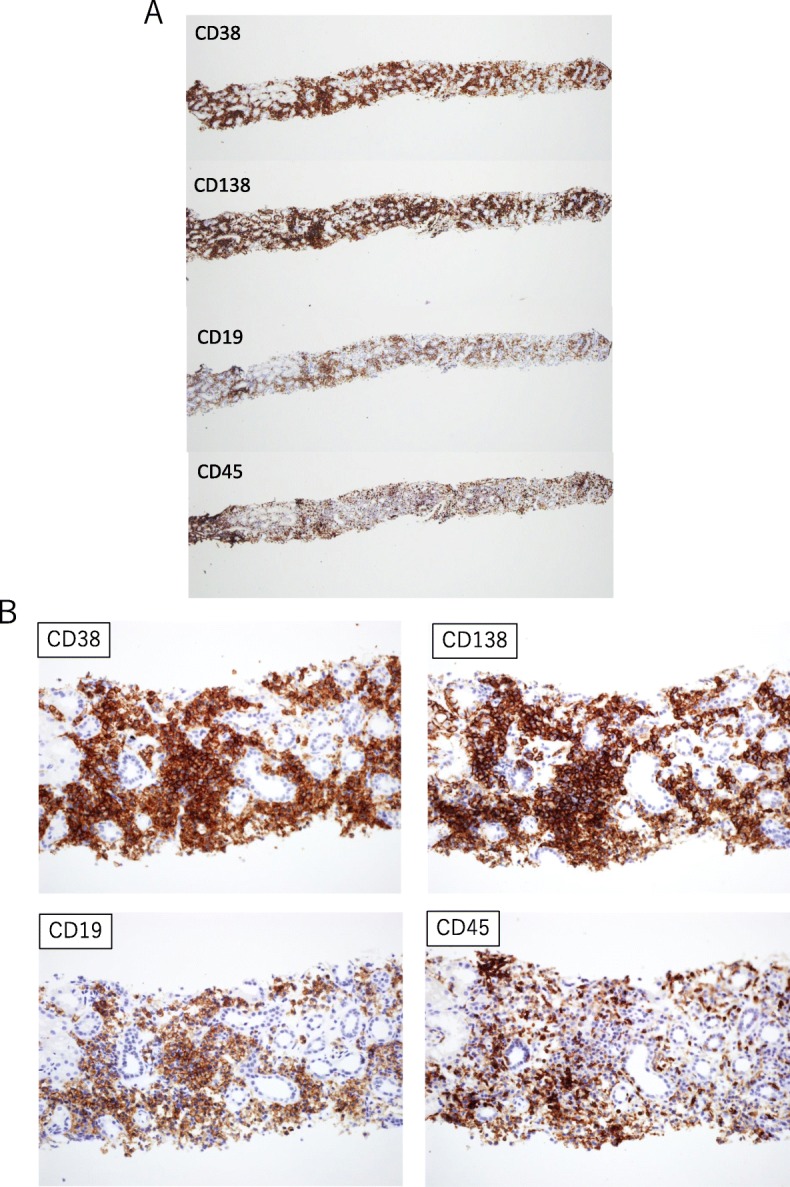
Immunohistochemical staining for CD38, CD138, CD19 and CD45 of renal biopsies. **a** × 40. **b** × 200

Renal biopsy demonstrated abundant plasma cell-rich mononuclear cell infiltration in the renal interstitium (Fig. 1). A few inflammatory cells in the basolateral aspect of the renal tubule epithelium were observed in some tubules. Among twelve glomeruli, three were globally sclerotic and the others showed minor abnormality, consistent with tubulointerstitial nephritis (TIN). Congo-red staining was negative for amyloid. Immunohistochemical staining for IgG, IgA, IgM, IgG4, kappa and lambda demonstrated that most of the infiltrative cells were positive for IgA and kappa (Fig. 2). Immunohistochemical staining revealed that most of the infiltrative cells were positive for CD38 and CD138, but cells positive for CD19 and CD45 were also widely evident (Fig. 3). IgG4 staining was negative. Immunofluorescence microscopy revealed negativity for immunoglobulins, complement, and lambda or kappa light chain on either the tubular basement membranes or the glomeruli. Electron microscopy revealed no deposits or crystal inclusions in the glomeruli and tubules. Infiltrative cells in the renal interstitium were mainly plasma cells and lymphocytes without atypia. Russel bodies and Dutcher bodies were not evident. Histological examinations of the salivary glands and lung were not performed at the patient’s request.

**Fig. 4:**
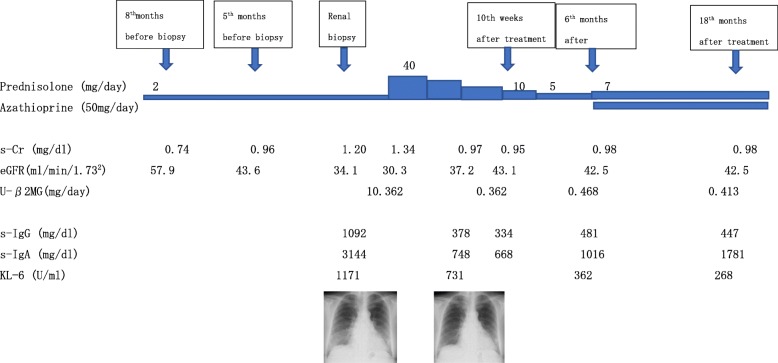
Clinical course of this patient. s-Cr; serum creatinine, U-β2MG; urinary β2-microglobulin, s-IgG; serum IgG, s-IgA; serum IgA

The dose of prednisolone was increased from 2 to 40 mg daily. The serum creatinine, eGFR and urinary β2-microglobulin level were immediately improved (0.95 mg/dl, 43.1 ml/min/1.73^2^ and 0.544 mg/day ten weeks after the start of therapy, compared with 1.34 mg/dl, 30.3 ml/min/1.73^2^ and 10.362 mg/day before therapy), and the bilateral lung reticular shadows were also immediately improved. Six months after the start of therapy, when the maintenance prednisolone dose was 5 mg daily, the serum creatinine level was slightly elevated (0.98 mg/dl, eGFR 42.6 ml/min/1.73^2^), and the serum IgA level was re-elevated. Therefore, the dose of prednisolone was slightly increased (5 mg daily to 7 mg daily) and azathioprine 50 mg daily was added. Thereafter, renal function was maintained at the same level for 18 months, although the serum IgA level continued to increase gradually (Fig. 4). At 18 months after the start of therapy, bone marrow re-examination revealed normocellular marrow and plasma cells accounted for 1.0% of the total. Cells positive for CD19 or CD45 were few, and mutations in the myeloid differentiation primary response gene 88 (MYD88) were not evident. 18FDG-PET/CT scanning performed 18 months after the start of therapy showed no FDG uptake including the lymph nodes and lungs.

## Discussion

In the present case, gradually progressive renal injury developed during the course of MGUS, and therefore MGRS had to be considered. Most MGRS-associated lesions are caused by deposition of monoclonal immunoglobulins in whole or in part, or aggregates of their various products [4]. This results in glomerulopathies with organized deposits such as AL amyloidosis, cryoglobulin nephropathy, non-organized deposits such as monoclonal immunoglobulin deposition disease (MIDD) or proliferative glomerulonephritis with monoclonal immunoglobulin deposits. MGRS also includes renal tubule disorders such as light chain proximal tubulopathy, or MIDD. However, the renal pathology in this case was plasma cell-rich TIN with no immunoglobulin deposition and no deposits/inclusions in the glomeruli or renal tubules, and in addition, the infiltrative cells were monotypic (IgA kappa).

TIN is sometimes evident in monoclonal gammopathy. However, most of such cases are monoclonal light chain-mediated and the infiltrative cells are not usually monotypic [2]. Rarely, interstitial nephritis has been described as neoplastic interstitial plasma cell infiltration. Attias et al. described a case of MGUS (IgG-kappa) with renal dysfunction [5]. Kidney biopsy revealed the presence of atypical multinucleated CD138+ plasma cells with voluminous nuclei stained exclusively with a kappa antibody. The patient was diagnosed as having indolent multiple myeloma with specific renal involvement in the form of malignant monotypic interstitial plasmacytic infiltration. However, in the present case, most of the infiltrative cells were positive for CD38 and CD138 without atypia. In addition, cells positive for CD19 and CD45 were also widely evident, resembling the cytologic features of lymphoplasmacytic lymphoma (LPL), but not plasmacytoma.

LPL is an uncommon B-cell neoplasm composed of small lymphocytes, plasmacytoid lymphocytes and plasma cells. The lymphocytes express the pan-B cell markers CD19 and CD20 and the plasma cells express CD138 and CD38 and often retain expression of CD19 and CD45, unlike myeloma [6]. Serologically, the majority of patients with LPL demonstrate monoclonal gammopathy. Monoclonal serum IgM is the most common subtype and confers a diagnosis of the clinical syndrome Waldenstrom macroglobulinemia. LPL usually involves the bone marrow, and sometimes the spleen or lymph nodes. The plasmacytoid lymphocytes and plasma cells contain abundant intracellular immunoglobulin, which is usually IgM, although rare cases may express IgG or IgA. In addition, in at least 90% of cases, the condition is associated with mutations in MYD88 [6]. In the present case, the immunophenotypes of the renal infiltrating cells were similar to those in LPL. However, the paraprotein was IgA, not IgM. There was no apparent hematological malignant involvement such as the bone marrow or lymph nodes, and mutations in MYD88 were not evident in the bone marrow. In this regard, the present case appears to be unique.

On the other hand, TIN is the most common renal manifestation of pSS [7, 8]. In TIN of pSS, predominant plasma cell infiltration is often evident, but the infiltrative plasma cells are usually polytypic [7, 8]. However, TIN with monotypic plasma cell infiltration has been reported in some patients with pSS accompanied by MGUS. In 2017, Saglam et al. reported 2 patients with pSS accompanied by MGUS who developed monotypic, but not atypical, tubulointerstitial plasma cell infiltration involving the kidney [9]. One of them was a 68-year-old female and the serum monoclonal paraprotein was IgA-lambda. The level of serum creatinine was 2.2 mg/dl and renal biopsy revealed interstitial infiltration of plasma cells, which were positive for CD138 with lambda light chain restriction. The other patient was a 69-year-old female in whom the serum monoclonal paraprotein was IgA-kappa. The level of creatinine was 2.3 mg/dl, and renal biopsy also revealed TIN with infiltration of plasma cells, which were positive for CD138 with kappa light chain restriction. In both cases, the plasma cells were positive for IgA. Immunofluorescence revealed no light- or heavy-chain deposition on either the tubule basement membranes or the glomeruli. The patients were diagnosed as having non-tumor-forming interstitial neoplastic plasma cell infiltrations. They received chemotherapy (melphalan-prednisolone for the former patient and bortezomib-dexamethazone for the latter) and the serum levels of creatinine thereafter decreased to 1.8 mg/dl and 1.1 mg/dl, respectively. In 2006, Kobayashi et al. reported a 49-year-old female patient with pSS and MGUS who developed TIN with Fanconi’s syndrome [10]. Also in that case, most of the infiltrative plasma cells were positive for IgA and kappa, and there was no monoclonal chain deposition or electron-dense crystal formation in the glomeruli or tubules. Steroid monotherapy (prednisolone 30 mg daily) resolved the renal dysfunction (serum creatinine 1.29 to 0.91 mg/dl). Unlike usual MRGS, in which the kidney lesions are related to the produced monoclonal immunoglobulin, infiltration of monotypic cells without immunoglobulin deposition is the main feature in cases of TIN and pSS accompanied by MGUS. Interestingly, the paraprotein in all of these reported patients, including the present one, was IgA. Although the pathogenesis of this renal involvement is unknown, some autoimmune mechanism might be involved, as has been shown in ordinary TIN in pSS. Further accumulation of similar cases will be necessary.

The renal function in the present patient improved rapidly upon administration of prednisolone at 40 mg daily and has been maintained at the same level on low-dose prednisolone and azathioprine for 18 months. Because lymphoproliferative disorders often respond well to steroid therapy, even some lymphomas may show similar responsiveness. However, if renal function deteriorates, it is necessary to examine the renal histology again for development of monoclonal immunoglobulin deposition or apparent hematological malignancy. In addition, changing azathioprine to another immunosuppressant might be necessary because the level of serum IgA is still increasing. We are now following up the present patient carefully.

In conclusion, we have presented a rare case of pSS with IgA-type MGUS in which tubulointerstitial nephritis developed along with monotypic (IgA-kappa) lympho-plasmacytic cell infiltration. Further accumulation of similar cases will be necessary.

## Data Availability

Not applicable.

## Abbreviations

CT: Computed tomography
eGFR: Estimated glomerular filtration rate
LPL: Lymphoplasmacytic lymphoma
MGRS: Monoclonal gammopathy of renal significance
MGUS: Monoclonal gammopathy of undetermined significance
MIDD: monoclonal immunoglobulin deposition disease
MYD88: Myeloid differentiation primary response gene 88
PET/CT: Positron emission tomography with computed tomography
pSS: Primary Sjögren’s syndrome
TIN: Tubulointerstitial nephritis
